# The Effects of Seawater Treatment on Selected Coniferous Wood Types

**DOI:** 10.3390/ma16175831

**Published:** 2023-08-25

**Authors:** Kamil Roman, Mateusz Leszczyński, Seweryn Pycka, Witold Jan Wardal

**Affiliations:** 1 Institute of Wood Sciences and Furniture, Warsaw University of Life Sciences, 166 Nowoursynowska St., 02-787 Warsaw, Poland; witold_wardal@sggw.edu.pl; 2 Faculty of Wood Technology, Warsaw University of Life Sciences-SGGW, 166 Nowoursynowska St., 02-787 Warsaw, Poland; s196970@sggw.edu.pl (M.L.); s190114@sggw.edu.pl (S.P.)

**Keywords:** strength, mechanics, stretching and compression of wood, stresses

## Abstract

The mechanical strength of wood from Scots pine (*Pinus sylvestris*), European larch (*Larix decidua*), and Norway spruce (*Picea abies*) was studied using static compression tests. The material was exposed under constant soaking in water with salinity of 7‰. The liquid mix was prepared according to a value roughly equivalent to the average salinity along the entire length of the Baltic Sea. The mechanical strength and quality of the raw material were determined using a sea salt saturation test, which determined the adhesion of the raw material to the extrusion process (permissible stress). An investigation was conducted to determine the physicochemical parameters of the material that was tested. It was investigated how much mineral compounds were absorbed over four cycles lasting a total of six weeks during the test. According to the statistical analysis, the chemical composition of wood and the presence of salts and mineral compounds correlated with its mechanical strength. An important part of the study focused on examining the factors affecting the construction of coniferous wood structures. The preparation of the raw material correctly can provide information on how the material can be protected during exposure to specific environmental conditions for longer.

## 1. Introduction

The use of wood in marine environments has been a topic of interest for many years due to its susceptibility to decomposition and degradation when exposed to seawater. Many studies have examined the effects of seawater on the durability and performance of wood in marine environments. “Wood and its Applications: a handbook for non-engineers” by Hsu and Hse [1] provides an overview of the properties of wood and its various uses. Gaff and Gunstone [2] discuss the effects of seawater on wood durability, while Evans and Michell [3] focus on choosing the appropriate wood for marine and freshwater construction. Basta and Hasanean [4] review the state of knowledge on the performance of wood and wood-based materials in marine environments. Mohd Ishak and Salim [5] emphasize the importance of wood properties in building wooden boats. Vér and Divós [6] study the effect of the immersion time on the mechanical properties of pine wood. Roffael and Militz [7] study the durability of heat-treated and untreated tropical wood against fungi and sea slugs. Widyorini, Hirai, and Imamura [8] study the weathering and leakage characteristics of wood treated with aqueous preservatives exposed to seawater. Deka and Saikia [9] study the effectiveness of wood preservatives in controlling sea slugs. Finally, Hapca and Petutschnigg [10] present the challenges and opportunities of using wood in marine applications.

Several factors play a role in the degradation of timber structures in the marine environment. These factors should be considered during the construction of such structures in and lying in coastal waters. Marine environments are some of the most challenging environments for materials, including wood, when exposed to them daily. Marine environments are notorious for their deterioration factors, such as perpetual moisture exposure and high humidity. These factors can cause wood to expand and shrink, resulting in dimensional changes and material weakness over time. The marine environment is also renowned for marine organisms, able to attack and degrade wood, such as aquatic borer pests, fungi, and bacteria. In addition, excessive exposure to sunlight can lead to the photochemical degradation of wood surfaces, characterized by color changes, surface checks, and the overall loss of strength of the wood over time. Several factors affect the integrity of marine structures, such as the leaching of wood preservatives from wood and the corrosion of metal fasteners in marine structures. Wood may experience frequent temperature changes, which can lead to its expansion and contraction, causing cracks and splitting as a result of this movement. To protect the wood in marine environments (UC5), it is necessary to understand and mitigate the factors that contribute to the degradation of the wood, such as moisture, biological attack, sunlight exposure, and saltwater exposure. It is possible to manage wood protection through a variety of separate activities.

- Degradation factors in the marine environment—To ensure that wood structures are durable in maritime environments, it is important to choose materials carefully, apply protective coatings, and provide regular maintenance to ensure their durability.
- Natural durability in UC5—To ensure the long-term durability of UC5 applications, it is recommended to select wood species that are exceptionally durable or softwoods that are highly rot-resistant. Due to the presence of natural substances in these woods, they are less susceptible to decay and, therefore, can last longer in harsh environmental conditions.
- Biocidal treatment for UC5—It is very important to use biocidal treatment for UC5 wood as it is prone to decay, making any treatment with biocidal agents crucial in enhancing its longevity and ability to perform properly. It is common for wood to be treated with biocide, including pressure-treated wood, which is treated under high pressure with preservatives so that they penetrate deeply into the wood.
- Modification treatment for UC5—A modification treatment involves altering the structure of the wood at the molecular level to increase its performance characteristics over time, including the ability to resist decay and resist moisture absorption. A combination of these treatments can be an excellent alternative to traditional biocidal treatments and in some cases can even be used for species of wood that are not so durable by increasing their natural durability.

Traditionally, wood quality testing involves a series of mechanical tests, such as measuring bending strength, compressive strength, and hardness. These tests are used to determine the material’s physical and mechanical properties. Furthermore, potential defects in the wood can be evaluated by examining it visually for knots, cracks, snakes, or other types of defects that could affect its strength and appearance, as well as its durability. As well as specifications for quality parameters for diverse wood applications, some norms and standards specify how to measure and evaluate quality parameters for these applications. The Australian Guidelines: Timber Service Life Design Guide [11] presents some practical recommendations when working with wood in contact with the ground. The document contains details on how to select timber species, grades, and treatments based on the environment and the intended use of the structure. This is to select the most appropriate timber species, grades, and treatments. There was a detailed analysis of how timber structures should be designed to withstand specific loads. This was performed considering factors such as the dead load, live load, wind load, and seismic load. The recommendations regarding treatments, storage, sustainable forestry practices, life cycle assessment, managing moisture, timber’s fire resistance, and fire safety structures were also considered. The specific guidelines and regulations are likely to differ depending on the country or region, as building codes and standards might differ from one area to another. The importance of using the appropriate wood in the testing process is crucial to achieve the desired results and to ensure the safe and effective use of this material in the context in which it is used.

Wood sorption is the ability of a material to absorb or release moisture from the environment, depending on the air humidity. Lesar et al. researched the sorption, durability, and mechanical properties of wood treated with NaCl [12]. The work by Lesar and co-authors focused on the study of wood sorption properties after salt treatment (NaCl). The research determined the effect of NaCl on wood’s sorption capacity and compared the wood’s sorption capacity before and after salt treatment. It also determined the possibility of dimensional changes in wood related to its ability to absorb moisture after salt treatment. Melcher’s research [13] focused on assessing wood durability after salt treatment (NaCl). Wood durability refers to its resistance to biological decay, such as attack by fungi, insects, and rotting organisms. During the analyses, changes in wood resistance to biodegradation after salt treatment were evaluated, and the wood resistance before and after NaCl application was compared. Research by Brischke and co-authors [14] concerned wood treated with NaCl and its mechanical properties. Wood’s mechanical properties refer to its strength, flexibility, and load resistance. The test results contained valuable information about salt treatment’s effect on wood strength. The mechanical properties of the wood before and after NaCl use were also compared. In addition, possible changes in wood elasticity after salt treatment were evaluated. A review of these three studies can provide an understanding of the effects of salt (NaCl) treatment on various aspects of wood, including the sorption capacity, durability, and mechanical properties. These studies are critical for a better understanding of NaCl use in wood treatment and its long-term use under various environmental conditions.

In this paper, three of the most popular Polish coniferous wood species—pine (*Pinus sylvestris*), larch (*Larix decidua*), and spruce (*Picea abies*)—are considered. According to the authors of [15], one of the largest drawbacks of wood as a raw material, used in a wide variety of industries, is its susceptibility to the environmental and weather conditions in which the wood grows. Moreover, it is affected by the environment and climate of the site where it is used [16]. As a result of their action, the physical and strength properties deteriorate, so that the durability of an object composed of unprotected wood is also reduced. Unfortunately, the proper protection of wood against the factors that cause its degradation requires knowledge from many different scientific fields, such as physics, chemistry, construction, and biology [17], so that the person responsible for the protection of wood has sufficient knowledge to use the appropriate measures for the type of factor to which the wood is exposed. Correctly assessing the impact of the site conditions on wood is particularly important for wood used in the construction and boatbuilding industries, where weakening in its strength can lead to catastrophic accidents, such as the collapse of a wooden floor or deck. It is important to realize that the period of wood use until the end of the shipbuilding era spans thousands of years, but almost no value, feature, or advantage has remained constant for such a long period [18].

The use of wood for demanding construction applications has been on the rise in Europe for many years. A common type of wood used in wooden constructions is conifer wood, which is susceptible to wood decay due to fungi in wet environments [19]. To ensure its service life, most wood must be treated with preservatives [20,21]. The chemical treatment of wood can, however, change its mechanical properties. As a result of research [22], it was clearly demonstrated that wood preservatives do not significantly affect its mechanical properties, except in cases of untreated copper–ethanolamine and boric acid wood, which exhibited significantly lower structural integrity. Through exposure to liquid water or high relative humidity, it was found that the negative effects on strength and structural integrity could be reversible [22]. Some studies [23] have been conducted on the hazards associated with wood in seawater, regarding whether it is below or above the water level. An assessment of wooden constructions partially exposed to seawater in practice, based on field studies and observations, was provided. In this study, the main focus was on the effectiveness of sodium chloride (sea salt) against basidiomycetes in the laboratory and, in this context, how sodium was distributed in roundwood after exposure to artificial seawater. In the chemical analysis, it was found that there was sodium content of approximately 25 g per square meter 20 cm above the water level. It was found that 40.000 ppm was quantified over a pine log cross-section, significantly higher than the threshold value of 15 kg/m^3^ [23].

The novelty of our research in the topic of NaCl-treated wood is the application of modern methods and technologies, which allows an improved understanding of the mechanisms occurring during this process and the application of these methods and technologies in a way that is more environmentally friendly, efficient, and cost-effective. To improve wood’s durability and mechanical properties, it has been shown that NaCl absorption, which fills the intramolecular pores within a molecule, is a method that enhances NaCl absorption. In conjunction with the use of advanced analysis methods such as X-ray imaging (XRD), this method will permit the study of the NaCl treatment process under real conditions, allowing the investigation of the changes occurring inside the wood under real conditions and enabling a better understanding of the changes occurring inside the wood. Several parameters can be optimized for the NaCl treatment process (such as the salt concentration, time, and temperature) to improve the understanding of the process and maximize its benefits. Further studies might focus on combining the NaCl treatment with other factors, such as the wood species. This might result in a synergistic effect, resulting in an increased degree of durability for wood. The application of NaCl treatment to wood has been used for thousands of years to improve wood’s durability and fire resistance, but there is an opportunity to apply the properties of treated wood in a new and innovative way by creating new applications based on their properties. Research aimed at developing new applications of the process that can greatly extend our understanding of the processes and maximize their potential in a modern context is essential in exploiting its potential.

## 2. Methodology

### 2.1. Material

Different types of wood are used during construction based on the intended use of the building. Wooden structures exposed to seawater need frequent regeneration with replacement materials. The wooden substitutes are usually diverse, which causes the analysis of samples from different raw materials. The above assumption requires raw test material samples to be analyzed with different wood species. The study used wood from the Scots pine (*Pinus sylvestris* L.), the European larch (*Larix decidua*), and the Norway spruce (*Picea abies*) families, representing the most common construction materials in Poland. There was a certain amount of wood harvested from a coastal strip, which was located approximately 50 km from the shoreline (Gościno Forest District, Debica Forestry). The climate zone lies in the southern part of Poland, with a transitional marine and terrestrial character, where the average precipitation per year is 600 mm. Several climatic factors have created an environment that is suitable for the growth of conifers and deciduous trees. In research on the use of wood, the quality of the wood has been considered to be one of the most important factors.

Many fields pay attention to wood characteristics, such as sapwood and heartwood, in areas such as construction, furniture, and the paper industry. The anisotropic nature of wood makes it difficult to determine its homogeneous strength, so it was assumed that the material was selected to standardize the statistical results due to its anisotropic nature. To select the most suitable wood for the project, both sapwood and heartwood, as well as the classification of wood quality, were taken into consideration. The heartwood of a variety of tree species was selected and taken from the center of each tree, the middle part of the tree, and was collected as individual samples. The structure of beech wood is characterized by a higher density and a harder cellular structure, which makes it more durable and less prone to being damaged in the future. In the study, the heartwood of the trees was used because this part of the wood is more frequently used in wooden structures, especially those that require a high level of strength. There were faint defects visible on the plane of the wood, which were not detrimental to the strength and usability of the wood, allowing it to be classified as class B, based on the slight defects that were visible on the plane of the wood.

The species of pine, spruce, and larch are three of the most important wood species in Europe, and they are of significant global importance. There are several reasons that they are vital from a global perspective, and this is mainly due to the wide variety of applications that they have in the construction, furniture, and paper industries, as well as their availability and economic value. Several countries around the world depend on pine, spruce, and larch as valuable wood resources and raw materials. Additionally, these species of wood are in great demand in the world market due to their beneficial properties and attractive appearance. There is a high demand for pine wood in both the construction and furniture industries, making it one of the most important types of wood in the world. It is popular for building wooden structures, floors, windows, doors, and furniture because it has good mechanical properties and is relatively easy to process. A major wood species in the global timber industry is spruce wood. The use of spruce in construction, particularly in wooden structures, is popular. With its high quality and durability, larch is a favored wood species for both interior as well as exterior applications, as it is attractive as a veneer material. This durable wood is also used for bridges, fences, parquet floors, and other structures that require durable wood, due to its natural resistance to biodegradation.

### 2.2. The Material NaCl Treatment

The study involved comparing the wood’s properties to those of the native material after cycle NaCl modification. It was necessary to place the samples in a solution of sodium chloride to prepare them for testing. Taking into account the salinity of the Polish part of the Baltic Sea, this corresponded to very salty water with average salinity of approximately 7‰. The samples were prepared in three stages by soaking them in water for varying periods. The preparation of the solution for the cycle required it to be left out for two weeks. Cycle II needed to be left out for four weeks, and cycle III needed to be left out for six weeks. To prevent crystal formation on the surface of the treated wood after soaking in the NaCl salt solution, it was necessary to take into account the intended purpose of the soaking. At a low solution concentration of 7 g/L, the wood was soaked. When the wood was soaked in an unsaturated salt solution, it reduced the likelihood of crystals forming on the surface of the wood. To achieve 12% moisture content in the prepared samples, the samples were stored in an air-conditioned chamber after soaking for the specified time [24]. 

The volume of the liquid that was absorbed during immersion was estimated based on the mass of the sample. Studies were developed based on a previous analysis that was already published in the literature [16]. The moisture before the strength test was always 12% [24] for comparison purposes. The moisture content of the samples was measured according to the PN-ISO 589:2006 standard [25]. It is possible to develop further studies by changing the moisture content. This can affect the moisture content (MC) and the growth of NaCl crystals in wood and its impact on the mechanical properties. However, the material strength tests were aimed at maintaining the same moisture content concerning the standard [24], while the issue of NaCl absorption was demonstrated in the ash measurement tests, to be more widely dissected in the authors’ previous article [16]. The weighted vessels were dried in a dryer at 103 °C during this study. Once the containers were removed from the dryer, they were cooled down in a desiccator for 30 min. It was decided to place the wet materials into the empty dishes one by one to weigh and measure the filled containers. This was performed after measuring the empty dishes used. The weight of the materials was then read to determine the weight of the wet material. It was decided to place the weighing vessels as well as the material into a dryer at 103 °C to dry them. An electronic scale was used to determine the weight of the dry material.

The homogenization of the sample is essential to ensure reliable and accurate results for ash testing. In ash testing, the inorganic residue is evaluated after a sample is combusted at high temperatures. This is performed to determine the amount of organic residue that remains. Random sampling was used to collect a representative sample of the entire testing lot. Because the samples had a solid structure, the preparation procedure was more demanding since they had to be milled into smaller pieces. Mechanical blend techniques were applied during homogenization to ensure equal sample mixing. There were no problems when shredding large material samples with a 1 mm^2^ shredder. Every prepared sample was supported by quality control and traceability, and samples were divided by species to achieve this. To ensure that samples were properly stored, airtight containers were used. 

### 2.3. Density Profile 

To characterize the wood modified in the sodium chloride solution, one of the most vital investigational elements was density profile analysis. Based on the research assumptions, the process may have altered the structure of the material. As a result, the physical and mechanical properties, such as the density, of the material may have been impacted as well. To understand how far the modification of the wood affected the internal structure and density of the material at various places in the sample, it was necessary to analyze the density profile of the wood before and after modification. The objective of the study was to determine the extent to which the structure of the wood changed regarding the intensity of the modification process used. In the methodology, the wood samples had to be technologically prepared for testing. To provide measurement dimensions for the sample profile, it was required to cut the profile to 500 × 500 mm. To measure the height, width, and thickness dimensions of the prepared sample, we used a caliper, ensuring that they corresponded with the recommendations of the manufacturer.

**Figure 1:**
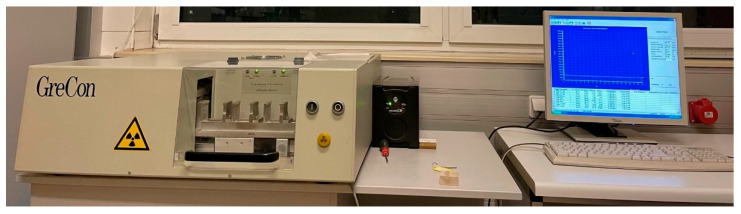
The test stand for density profile measurements.

The measurement was performed using the GreCon device (Fagus-GreCon, Hanover, Germany) an X-ray densitometry device, to determine the wood density profile. An attached frame is included in the device, on which wooden samples can be placed for testing. To measure the density of the wood, an integrated measuring head is moved along the sample, to analyze the density of the wood at specific points along the way. The X-ray densitometry method was used to determine the intensity of the transmitted waves of a mounted sample in the range of approximately 0.01 nm to 10 nm. The passing of an X-ray beam over the sample to analyze the intensity of the transmitted rays was necessary. This measuring head was equipped with an X-ray emitter and detector, so it was capable of detecting the waves passing through the wood sample as they passed through the X-ray emitter and detector. The wood density profile was then generated by specialized software in the form of graphs or spreadsheets based on the data generated. GreCon’s density profile test rig is presented in Figure 1.

The results from X-ray densitometry are presented in the form of a radiograph and provide information about the density distribution along the length of the sample cross-section as a function of the length of the cross-sectional area used. Using an X-ray image, the user is capable of extracting the density profile of a wood sample, which is presented as a visual representation on the X-ray image. The image can be analyzed to obtain basic statistical data related to the density of the area in question as a result of the analysis. Based on the measured data, it is possible to digitally characterize and compare the density profile for a selected species of wood in comparison with a prepared modification of the wood. There is one major advantage of X-ray densitometry, which is the fact that it is not a destructive method of measuring materials. The density distribution of wood samples can be a useful tool in characterizing the structure and properties of wood by providing information about the density distribution of wood samples. To interpret the results in the context of the research problem, it was necessary to compare the density profiles of selected wood species, by comparing their modified forms to a native standard to determine meaningful information. Based on the density profiles of the prepared samples, it was possible to predict the behavior and properties of the material under investigation. The analysis of density profiles requires the application of experimental techniques, the analysis of data, and the interpretation of the results. The purpose of this is to gain a better understanding of the material structure before and after it has been modified. 

### 2.4. Compressive Strength Tests 

**Figure 2:**
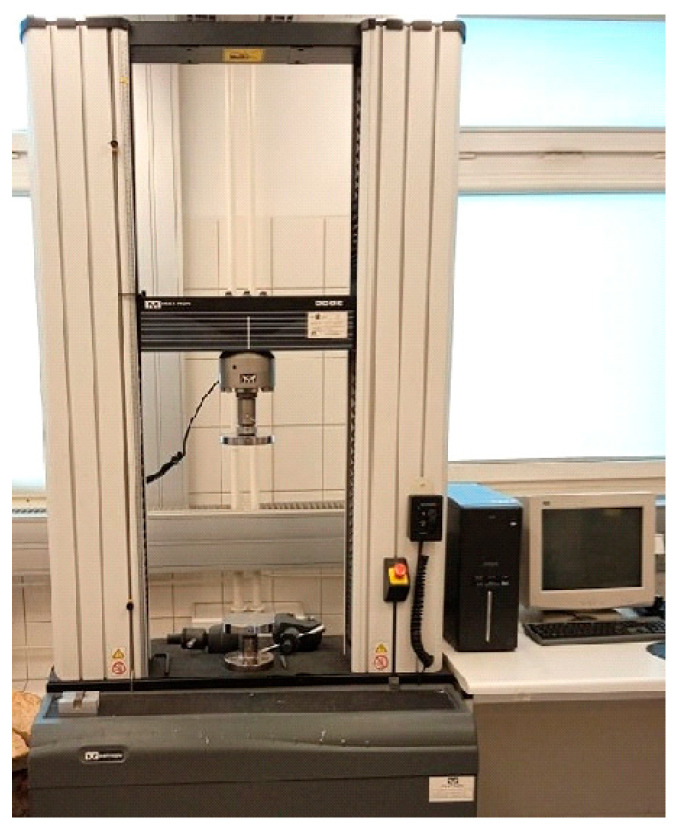
The test stands with attached dishes for compression samples.

To test the pretreated samples along the fibers, the samples were compressed using an Instron 3382 testing machine (Norwood, MA, USA) located in the laboratory. During the incineration process, the number of minerals absorbed by the materials, after they have been subjected to strength tests, can be determined by the amount of minerals absorbed by the samples. To determine the relationship between the material strength parameters and the soaking time, a comprehensive analysis of the samples should be conducted. The context of this is the absorption of salt in the body. The preparation of a suitable test stand was necessary for the planned strength tests to be carried out. Several components composed the test stand, including a machine that was used to perform compression tests, plates with compression test marks, and a computer that was used to measure data. The test stands with the attached dishes for compression samples are presented in Figure 2.

The measurement of the compressive strength along the fibers was conducted under the measurement procedure recorded in PN-D-04102:1979 [26]—Wood—Determination of the compressive strength along the fibers of the wood. To measure the density of coniferous and deciduous wood samples, a caliper was used in the measurement process. According to the standard, the dimensions were controlled as height h = 30 mm and width of the transverse section a = 20 mm and b = 20 mm in the transverse section, as recorded in the standard. Testing was carried out on a machine that dispensed loads ranging from 0 to 100 kN. Static testing was performed at a speed of 5 mm per minute. The failure force *P* [N] was determined after the measurements were taken, the value of which was divided by the cross-sectional area of fibers a and b [mm^2^] to determine the compressive strength along the fibers, which correlated with the cross-sectional area.

According to the strength tests, the prepared samples were examined to determine their strength. As part of the strength test studies, the Instron machine 3382 (Illinois Tool Works Inc., Chicago, IL, USA) with a suitable attachment was used. There was a computer connected to the testing machine as part of the measuring stand to perform the testing. The purpose of the study was to determine the compressive strength of prepared specimens using a compression test [27,28]. Based on the results of this test, it is possible to determine the failure stress, yield strength, and strain energy of a specimen from the characteristics that are measured [27]. The static compression test was carried out under the following PN-D standards: PN-D-04102:1979 [29], PN-D-04229:1977 [30], and PN-D-04115:1958 [31]. Compressive strength is determined by the direction in which the materials are applied and the resistance that they create. There can be significant differences in compressive strength for anisotropic materials due to their anisotropy. It is necessary to use standard-shaped samples of wood to determine their compressive strength.

### 2.5. Measuring Mineral Values

**Figure 3:**
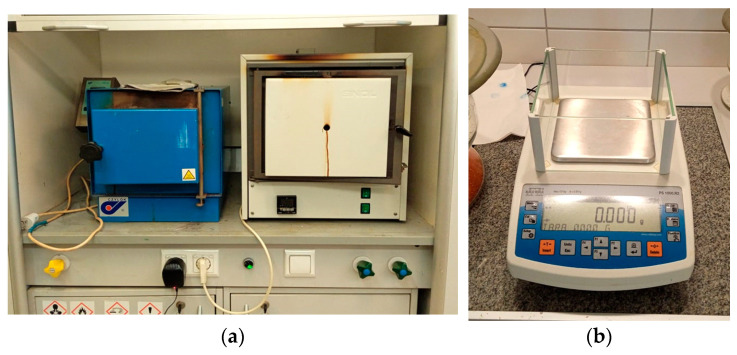
The laboratory test stands: (**a**) muffle furnace; (**b**) laboratory scale.

In the course of the study, we ashed the samples to characterize the absorption of sodium chloride in the material under study. This is related to other microelements’ absorption. During thermal decomposition, wood contains microelements that act as catalysts for the oxidation of gases formed during combustion. This results in ash, a fine-textured substance. Under this assumption, the difference between the ash content of the raw material during the soaking process and the ash content of the native material is due to the mineral saturation that takes place during the soaking process. In addition to microelements, other macroelements have not been identified. By using a muffle furnace, it was possible to measure the amount of ash contained in the sample. During ash content measurement, the raw materials must be dry. The test was carried out by placing a weighed sample of 2 g into the crucible and preparing the sample for testing. Triplicate measurement was performed with 0.0001 g accuracy. During the burning process, a muffle furnace of 805 °C was used. The SNOL’s muffle furnace (SnolTherm, Narkunai, Lithuania) and the Radwag (Radwag, Radom, Poland) Company laboratory scale that were used in this experiment are presented in Figure 3.

According to the methodology, approximately two hours later, the samples’ ash content was measured after preparation. To cool down the crucible, the ash sample was placed in a desiccator for 15 min. The percentage of ash in the sample was determined using the weight method, under Technical Specification PN-G-04512:1980 [32]. Based on the absorbency of sodium chloride, among the other microelements present in the test material, we determined that percentage that was absorbed. This was the difference between the weight of the raw material before obtaining the ash and the weight of the crucible and any contents of the crucible. The crucible content was compared with the raw material’s weight before the ash was obtained. The results of this research were based on previous analyses of wood that had been soaked in NaCl, and we compared and described the results from the table with those found in the literature [16]; the preparation of the tested samples and methodology were also described.

## 3. Results

### 3.1. Density Profile 

To measure the wood’s density profile, an X-ray scanner scanned the prepared wood samples. The tool used an advanced measuring device, designed to allow accurate and fast characterization of the density profile along the entire length of the sample. To measure the density of wood, X-rays, which is a non-invasive and accurate method, were used. To evaluate the modified nature of the wood structure, it was necessary to compare it with the reference sample, which had been prepared beforehand. It was possible to accurately determine changes in the internal structure of the wood, as well as the density of the wood, after modifications were made, using this measurement. The alteration process had the effect of modifying the properties of the wood in a manner that characterized the result. The density profiles for the pine, spruce, and larch samples for the native (reference) materials and in cycles 1, 2, and 3 are presented in Table 1.

The standard deviation was calculated by measuring the difference between the mean and the data points, which helped us to determine whether the density values were consistent or spread out over a wide range of density values for each material and test. In each wood type and with each test, the values represented the dispersion of the density measurements around their respective means, which corresponded to the type of wood and the test. This information could be used to obtain an understanding of the variability in the density measurements of each type of wood within each of the test groups, leading to a better understanding of how consistent or inconsistent the values of density were for each type of wood, depending on the testing conditions. The standard deviation for every species of measured wood depending on the type and modification is presented in Table 2.

**Figure 4:**
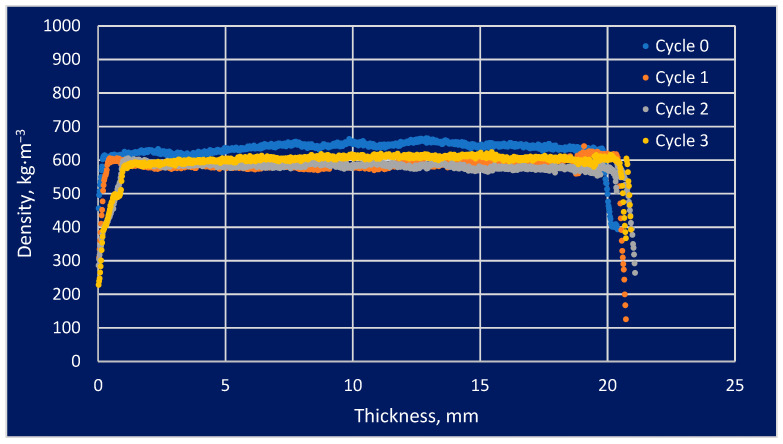
The effect of the number of soaking cycles on the density profile of pine wood.

**Figure 5:**
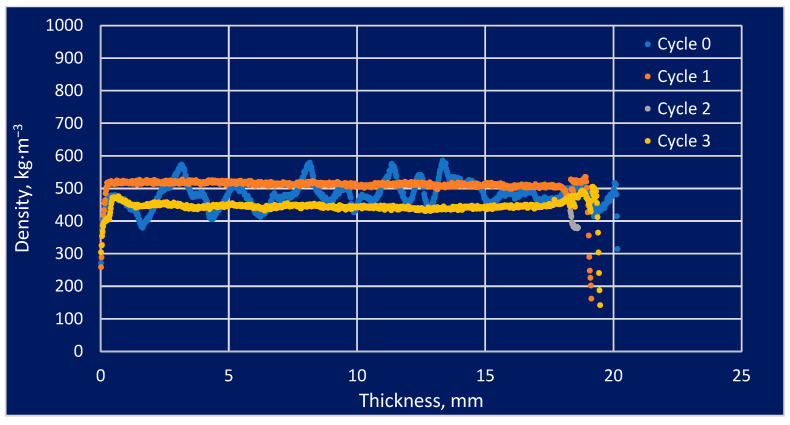
The effect of the number of soaking cycles on the density profile of spruce wood.

**Figure 6:**
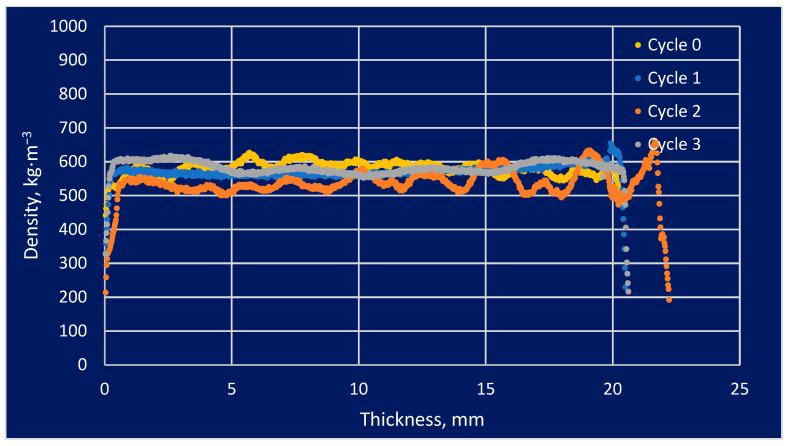
The effect of the number of soaking cycles on the density profile of larch wood.

The density profile study found that some areas that were more vulnerable to modification and its impacts had higher densities than areas that were less susceptible. This issue is important regarding the use of modified wood under varying weather conditions, including variations in humidity as well as temperature. The information provided by these sources allows for a more precise understanding of the properties of modified wood and how it could be adapted to specific applications. The analysis of density profiles is one of the most critical tools in the study of modified wood. The effect of the number of soaking cycles in the density profiles is presented in Figure 4, Figure 5 and Figure 6.

The density profile could be analyzed to determine how the modification processes affected the wood’s swelling and shrinkage. The graphical results above represent the material density profile concerning the sample thickness. This analysis was performed to provide a general idea of the absorption of salt solutions by the test material in a general sense. Variations in the density profile graph could be expressed in a variety of ways, not necessarily related to soaking cycles. Pine wood was characterized by a straight line, which indicated a constant density along an independent variable over time. The behavior of the substance indicated homogeneity in the substance or a uniform density distribution across a given area. There was a cyclic change in density along the sample thickness, which could be explained by the early cycles of spruce and larch. It may be that the absence of later oscillations was caused by salt filling the intercellular spaces in the cells.

### 3.2. Strength Testing

Scots pine (*Pinus sylvestris* L.) wood was one of the materials tested during the study. In terms of the natural characteristics of the wood, the material structure displayed anisotropic parameters. Wood as a natural material has very high hygroscopicity, which explains why moisture affects all of its properties to a great extent. The compression process, performed on a test machine with a plane for the compression of samples, was carried out at approximately 12% of the moisture content of the test materials. In the case of a change in moisture in the material, shrinkage or swelling may occur, which impacts the change in the dimensions of the material. Performing moisture control is an essential step in ensuring that the samples are measured correctly and that the mechanical properties of the samples are accurate. It was necessary to conduct additional inspections of the material, which helped to keep samples in the required condition.

Under the applicable norms, a static compression test was conducted and the results were listed in a table following the test. Under the standards that were designated, static compression and tensile tests were conducted. In technical terms, compressive strength refers to the resistance that a material provides to a force that may result in the deformation or destruction of the material. This is when the force applied is compressive. Wood is an anisotropic material, which means that the elastic moduli vary depending on the anatomical direction in which it is being used. There are differences in wood’s elastic modulus in its longitudinal, tangential, and radial directions, unlike in isotropic materials. It has been found that wood has the greatest strength in the longitudinal direction, followed by the tangential and radial directions [33]. There is a strong relationship between the modulus values of wood and the stiffness of the wood under different load conditions. To perform the tests, a machine with a compression speed of 5 mm per minute was used. The specimens were pre-cut to dimensions of 20 × 20 × 30 mm [34]. The tensile testing results of the native unsoaked material for pine, spruce, and larch samples are presented in Table 3.

The tensile tests along the fibers of pine, spruce, and larch as native materials without modification are summarized in the table above. The destructive forces, indicating the maximum force that the sample could withstand before breaking, and the tensile strengths along the fibers of each material were summarized. Based on the testing results of the pine material, the destructive forces ranged from 17.78 kN to 19.5 kN. The tensile strength of the material varied from 40.6 MPa to 47.2 MPa. Spruce’s destructive forces varied between 18.64 kN and 25.42 kN. There was variation from 43.9 MPa to 59.8 MPa in the corresponding tensile strengths along the fibers. It was estimated that the destructive forces in larch ranged from 23.06 kN to 24.65 kN and that the tensile strengths in the fibers ranged from 51.3 MPa to 56.8 MPa. When the respective fibers of these materials were subjected to tensile forces along their lengths, these results provided valuable information on the mechanical properties of their fibers. There was a general tendency in the data to suggest that the different types of wood differed in their strength and resistance to deformation.

In addition to the above, three different soaks of the modified material in salted water were also considered to compare the results. In the first case, tensile testing along the fibers was performed for cycle 1 pine samples, soaked in the prepared conditions for two weeks. The pine sample was tested at approximately 60 MPa, and the most significant value was 66.6 MPa, while the average value was 54.48 MPa. There was a large difference between the lowest strength, of 48.9 MPa, and the highest strength, of 63.2 MPa, for spruce. According to the results of the study, the average strength of all spruce samples was 55.3 MPa. There was a difference of 10.7 MPa between the lowest strength of larch and the highest strength of 56 MPa. Based on the results for all three larch samples, 49 MPa was determined to be the average strength. The results of tensile testing along the fibers for samples of the respective first-cycle materials are presented in Table 4.

The samples from cycle 2 of the fibers were also used for the tensile test. These samples were soaked in a special solution for four weeks to prepare them for the test. For pine samples, the lowest value was 39.3 MPa and the highest value was 50.7 MPa, according to the analysis results. It was found that pine samples averaged 45.6 MPa. According to the results for spruce, the lowest strength of the samples was 45.3 MPa, whereas the greatest strength was 60.3 MPa. Based on the samples, the overall mean strength was 54.4 MPa. The study of larch found that its lowest strength was 44.3 MPa, while its highest strength was 69.7 MPa. It was estimated that the mean strength of the research samples was 49 MPa. The results for all four-week-soaked samples in the tensile tests conducted across the fibers of the samples are presented in Table 5.

The next testing stage identified the tensile strength of cycle 3 samples, soaked for six weeks. It was found that the lowest strength of pine samples was 42 MPa, while the highest strength was 55.6 MPa. Samples of pine averaged at 50 MPa. In cycle 3, spruce samples had a minimum strength of 49.2 MPa and a maximum strength of 67.1 MPa. All spruce samples measured had a mean strength of 56.9 MPa. Larch had a minimum strength of 41.8 MPa and a maximum strength of 56 MPa. According to the samples of larch, the mean strength was 49 MPa. The tensile results along the fibers of the third-cycle materials are presented in Table 6.

**Figure 7:**
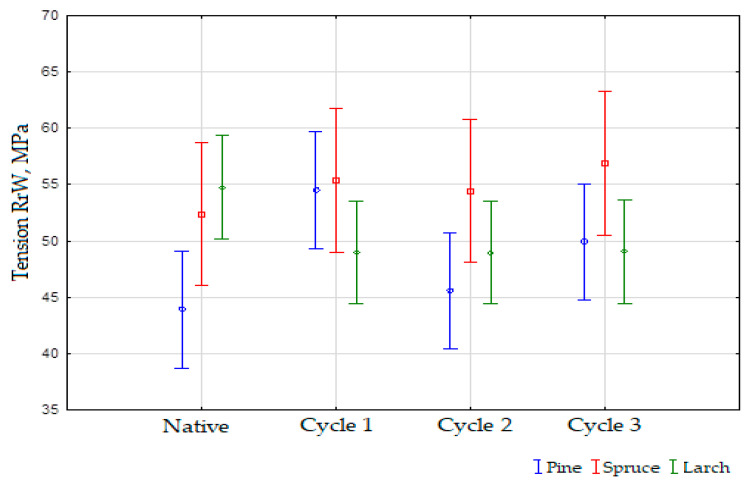
The effect of the number of soaking cycles used in saltwater on the compressive strength values of a given wood species.

The compiled results of the study were subjected to statistical analysis, which allowed the characterization of the results in the context of the effect of the number of soaking cycles used regarding the compressive strength values of a given wood species. Statistical analysis allows for the confirmation or rejection of the hypothesis that guides the authors during the research. The statistical analysis carried out showed the partial occurrence of differences between the measured parameters. The results led to the value of the degree of significance of *p* = 0.13132 for the empirical value of the statistic *F* (9, 34.223) = 1.6832. The average results of the analysis of the effect of soaking in saline water cycles on the compressive strength of each wood species are presented in Figure 7.

Statistical analysis of the pine strength parameters in a few cycles revealed that *p* was significantly greater than alpha 0.05, which was the assumed significance level. This means that the mean effects of the analysis of the number of soaking cycles on the tensile strength values for pine did not show any considerable differences. In this case, only one homogenous group was created with all the values for the pine soaking cycles. As for the statistical analysis of the strength parameters of the spruce samples prepared and tested in a few cycles, the level of significance *p* was higher than the assumed significance level alpha equal to 0.05. The effect of the number of spruce soaking cycles on the tensile strength values did not show any significant differences. Similarly, as in the earlier case, the post hoc test demonstrated only one homogenous group with all the least-squares means for the soaking cycles measured. In the last case of the statistical analysis of the tensile strength parameters of the larch samples tested in a few cycles, the level of significance *p* was higher than the assumed level of significance alpha equal to 0.05. A summary of the average effect of the number of soaking cycles used on the compressive strength values for all species is presented in Table 7.

Additional mechanical tests were conducted in a previous analysis that has been published by the authors in the literature [16]. Studies were developed and the results of the strength test were taken into account. The analysis consisted of a comparison between the results obtained from different strength tests carried out on three different types of wood. Wood samples included pine, spruce, and larch. According to the literature [16], for each of the materials, these tests included both tensile and compression tests. The compressive strength of pine was found to fluctuate between test cycles but, overall, it increased from the native values (43.90171 MPa) to cycle 1 (54.48221 MPa) and then decreased in cycle 2 (45.57737 MPa) and cycle 3 (49.89244 MPa), indicating a general upward trend. Similarly, the results of the compressive strength test of spruce also showed some fluctuation between periods, with the highest value in cycle 1 and the lowest value in cycle 2, with the highest value being 56.87965 MPa in cycle 1 and the lowest being 54.42522 MPa in cycle 2. Throughout the various cycles of the larch compression strength test, some fluctuations were evident, but, for the most part, the value remained at a similar level. The value for cycle 2 was the closest to the native value (48.95715 MPa), followed by cycle 3 (48.97611 MPa).

According to the analysis of the pine tensile test results, the tensile values appeared to be stable across the three cycles, with a slight increase from the native value of 82.5 MPa to cycle 3 with a value of 94.8 MPa. From the native values (98.8 MPa) to cycle 3 (113.8 MPa), the tensile strength of spruce was found to have increased significantly, indicating that the fiber strength of the spruce had improved significantly under cycle 3. There was a relatively high degree of stability demonstrated by the results of the tensile test of larch. The strength of the materials increased from the native values (77.5 MPa) to cycle 3 (95.27 MPa), which indicated a moderate improvement from the native values to cycle 3. Accordingly, there were marked differences between the different materials regarding the values of the tensile strength. Throughout the entire test process, spruce showed the strongest compressive strength of all wood tested. The tensile strength of spruce was also the highest. The strength of the larch wood was high, ranking between pine and spruce in terms of compressive and tensile strength. As the successive testing cycles progressed, spruce maintained a relatively stable strength, while pine and larch increased in strength. Based on the compilation of the test results, it was determined that spruce was the strongest wood material in both the tension and compression tests. A moderate improvement in strength was observed in pine and larch after successive tests were carried out.

### 3.3. Mineral Content

Under these assumptions, the measured mineral content of the raw material was regarded as a parameter. This information can be used to determine the degree to which wood can absorb a sodium chloride solution. The mineral saturation of the raw materials was determined by monitoring the amount of ash in the raw material and comparing it to the amount of ash in the native material after soaking. Several studies have investigated the percentage of ash content in pine, spruce, and larch samples in a variety of cycles over the years. To incinerate the samples over a sufficient period, the samples were placed in metal crucibles ranging in weight from 1.75 g to 2.36 g. The mean sample weight was 1.8 g. One by one, the samples were combusted in a muffle furnace by following the methodology and replicating the process several times. An analysis of the ash percentages in successive samples is presented in Table 8.

The effect of the salt solution on the ash content of the wood samples was an interesting aspect of the study. The results of the study are represented in the above table that lists the percentage content of ash in the given tree species in particular cycles. In this case, it was analyzed over a particular period. As long as the amount of ash content in the native material did not exceed 0.5%, there were no significant changes in the material. Similarly to pine, larch, which is native to the region, also showed ash content below 0.5%. The highest ash shares were recorded in cycle 1, and the overall highest ash share was recorded for pine, while the highest result for one of the samples was 0.9%. There was also a slight increase in mineral content in the other samples as well. It should be noted that, in cycle 2, the amount of ash in pine increased significantly and reached almost 1%. The mineral content of the other samples did not change from cycle 1 to cycle 2 and remained the same as in cycle 1. It was observed in cycle 3 that all the samples had similar mineral content, accounting for approximately 0.7% of the total mineral content. As a result of mineral supersaturation being recorded during cycle 2, the pine likely experienced a decrease in its mineral content. For the rest of the species, the supersaturation effect was already evident during the first cycle of treatment, or even at the beginning of the treatment.

## 4. Discussion

According to the results of the experiment carried out, we can conclude that the material that was used for the study had a certain influence on the structure of the wood species studied. There was a direct correlation between the way in which wood material is treated and the strength of the wood material, as well as the absorption of minerals. The type of effect that soaking had on the physical parameters depended on the species of wood examined, and the effects varied from species to species. As a result of performing density profile tests on the material with the introduction of the medium, we were able to characterize the absorption properties of the medium. This study was designed to determine how the modification of the tested material would affect its structure and how it could be improved. According to the density profile, for the most part, it had a straight or slightly sinusoidal structure with a relatively small number of curves. It was possible to observe peaks, depressions, or jumps in a minor part of the density profile. Such variations can arise from the concentration of substances within the system itself or as a result of interactions between various parts of the system under study. It was observed that the density profile was sometimes subjected to non-monotonic changes, i.e., there was an increase or decrease in density in different areas depending on the area. A wide variety of factors can contribute to the varying density of a material, including varying environmental conditions, chemical reactions, and competition between several factors that affect the density of a material. During the research, growth rings were not measured to determine how they influenced the outcomes of the study. This was because the measurement machine’s settings were not suitable for such measurements. The density varied depending on the tree’s age throughout the annual rings, as dictated by nature. According to the X-ray analysis of the spectrum, it was evident that the entire structure of the examined solid material was characterized by a different distribution of early and late wood. There was an assumption made at the beginning of the research that we would obtain the widest possible range of dependencies to examine in the course of the research.

To develop the research, it was also necessary to analyze the structure of the modified material in a mechanical context. The research that was conducted was illustrative and explained how the modification used in the study affected the wood species of interest. The wood was also tested for its compressive strength by performing static compression tests on the specimens. Larch wood was the only wood type with a decreasing trend, with a mean difference of 5.8 MPa. The average strength of the native samples was the highest in all of the samples, with an average value of 54.8 MPa. In cycles 1 to 3, the average strength of the wood was 49 MPa, which was one of the most interesting aspects of the study. There were positive average values for both pine and spruce in each of the cycles where these wood species were examined. There was a gradual increase in the strength of spruce. This began at 52.7 MPa and gradually increased until the peak was reached at 56.9 MPa at the end. There was an average strength difference of 4.2 MPa between the two species. It was discovered that pine had strength of 43.9 MPa when tested on the native material. In cycle 1, the results increased dramatically, but in cycles 2 and 3, they returned to their original levels and then the values increased once more. This was most likely due to the wood’s concentric compaction. The study presented in this manuscript was based on a previous study conducted on wood soaked in NaCl for several days. This wood was used for a tensile and compression study. The results from both sources were compared to obtain a better understanding of them [16]. For each material, these tests included both tensile and compressive tests.

Based on the results of the tests, it was observed that the wood’s compressive strength fluctuated throughout the testing period. From the native values (43.9 MPa) to cycle 1 (54.5 MPa), pine’s compression strength increased. However, it decreased in cycles 2 (45.6 MPa) and 3 (49.9 MPa), showing an overall upward trend. A similar pattern was observed in spruce regarding its compressive strength, reaching its highest value in cycle 1 (56.9 MPa) and its lowest value in cycle 2 (54.4 MPa). Throughout the various tests, on the other hand, the compressive strength of larch remained relatively constant throughout most cycles, with only a few minor fluctuations throughout. After cycles 1, 2, and 3, the value for cycle 2 was the closest to the native value (48.96 MPa), followed by cycle 3 (48.98 MPa). A stable value of tensile strength throughout the various test cycles was demonstrated for pine. There was a slight increase in the native values from 82.5 MPa to 94.8 MPa during cycle 3, owing to a slight increase in the native values. The spruce’s fiber strength showed a significant improvement under cycle 3 (113.8 MPa) compared to the native values (98.8 MPa). The tensile strength of larch increased moderately from native (77.5 MPa) to cycle 3 (95.27 MPa). Both the compressive strength and tensile strength tests showed spruce to be the strongest wood material. As well as demonstrating the greatest compressive strength, it showed the greatest tensile strength throughout the testing process. The compressive and tensile strengths of larch were the highest compared to pine and spruce. Pine and larch showed strength improvements as the test cycles progressed, while spruce maintained relatively stable in strength. In both the tension and compression tests, spruce was found to be the strongest wood material. The strength of pine and larch also improved moderately after successive tests were conducted, which was in agreement with the other findings of this study. The findings of this study can have important repercussions for the construction, engineering, and furniture-making industries, which use wood due to its mechanical properties.

In light of the research that has been conducted, it would also be interesting to analyze the mineral composition of the material. It would be useful to collect this information to indirectly assess the impact of material modification on compressive strength. The ash of the wood could contain several minerals that have been absorbed by the wood. It may be the case that a structure with high mineral content can absorb an increased proportion of saltwater. A further approach may be to determine the ash composition to predict how strong the material will be when compressed. Correlations provide fundamental information needed to perform an ash composition analysis and, based on this information, we could apply models to predict the compressive strength as a result of the analysis. Based on analyzing the results of the ash content test, it can be seen that the lowest percentage of ash was found in the native material in all three grades. It is important to note that although it increased in subsequent cycles relative to the native material, there was always an increase in this parameter. According to the species, the proportion of the total share is different, also depending on the cycle in which it occurs. For pine, it is the second cycle; for spruce, it is the first cycle; and for larch, it is the third cycle. Only one sample from each species and each cycle exceeded the value of 1 percent, namely at the second repetition for the spruce sample taken from cycle 3, which showed the highest content by far. The percentage of ash found in the remaining samples ranged from 0.34 percent in the native material samples up to values around 0.9 percent in the later cycles of soaking in saltwater, indicating that ash constituted a significant part of the composition.

## 5. Conclusions

This study examined the relationship between wood’s structure, modification processes, mechanical properties, and mineral composition, to illustrate the relationships between these variables. These findings can help us to better understand how wood materials behave and how they can be applied to other types of projects, in addition to gaining insights into wood’s physical, chemical, and mechanical properties. Furthermore, we have provided information about potential applications for wood that could be developed in the future as a result of the study. To investigate wood’s absorption properties and variations in density, the density profiles of wood were analyzed using X-ray scanning. The analysis of the density profiles of wood using X-ray scanning is a highly effective tool in determining wood’s absorption properties as well as identifying the density variations in the wood. Many factors can influence these variations, including the environmental conditions, chemical reactions, and other factors. The results of the study demonstrated that a variety of treatments had a significant impact on the wood’s structure and density. The effects of these treatments varied according to the species of wood that was used. Modifications made to wood can have adverse effects on its structure, strength, and ability to absorb minerals. In the study of the density profiles of wood, it was found that wood that had been modified had a higher density than wood that had not been modified.

Wood’s exposure to various weather conditions can cause negative changes. For this reason, the study also included a structural component, namely evaluating the compressive and tensile strength to determine the wood’s durability. There was a variation in strength among the different species and modifications of wood as a result of the test. With changes in moisture levels, there was a significant impact on the wood’s mechanical properties. There was a significant amount of shrinkage and swelling caused by changes in moisture levels in the material structure. Based on the results of the tests carried out, it can be concluded that wood, after being soaked in seawater, has improved mechanical properties. However, it does not deviate significantly from the Polish standard mechanical properties. The strength of pine wood was one of the more interesting aspects of this test. It was the lowest of all the species tested here, regardless of whether they were native or not. It was not apparent that the samples themselves were grainier or had more visible defects than the control samples. The positive results of the tests can also be attributed to the frequent water changes and agitation of the water to avoid salt crystal sedimentation. The moisture before the strength test was always 12%. Further research could examine how changing moisture may affect material strength tests aimed at maintaining the same moisture as the standard [24].

The ash content of wood, which is a measurement of its mineral composition, plays a significant role in the quality of the wood, as it impacts the compressive strength, with higher mineral content potentially resulting in the increased absorption of saltwater and stronger materials. The ash content of wood, as well as the mineral composition of the wood, can provide insights into its compressive strength. According to the results of the soaking cycles, the ash content increased, indicating higher mineral content than before. There was a considerable increase in the amount of ash in pine in the second cycle, reaching almost 1%. As a result of the ash analysis, an almost 200% greater change was found in the samples than in the native samples. There was a significant decrease in mineral content in pine during the second cycle of its growth, possibly as a result of mineral supersaturation. It was also found that other species showed a supersaturation effect from the first cycle of treatment onward.

Based on the data collected, the novelty of this study lies in the investigation of the relationship between wood’s structure, modification processes, mechanical properties, and mineral composition. There is an interrelationship between these variables. Moreover, the study explored how a variety of treatments can influence the structure and density of wood. The entire group of research [16] provides insights into the potential applications of wood based on the findings. Our work included an assessment of the wood’s absorption properties and density variations, which could be measured using X-ray scanning, an effective tool for the measurement of these properties. A study was also carried out on the compressive and tensile strengths of the wood to determine its durability in various environmental conditions, including seawater, and the effects of climate change. The results of the study revealed how the presence of ash and the mineral composition affected the compressive strength of the wood differently based on the wood species, as well as how modifications and treatments affected their properties. These outcomes are particularly significant in the context of saltwater absorption. Through its findings, this study contributes to the understanding of wood’s behavior and the potential applications of wood materials in a wide range of construction applications.

## Figures and Tables

**Table 1:** The results of wood density profiles in pine, spruce, and larch samples.

Type of Soaking	Material	Mean Sample Dimensions (SD) ^1^, mm			Mean Weight (SD) ^1^, g	Mean Sample Density (SD) ^1^, kg⋅m^−3^
		Height	Width	Thickness		
Native material	Pine	49.82 (0.34)	41.05 (0.19)	20.63 (0.05)	26.69 (0.04)	632.51 (3.92)
	Spruce	40.35 (0.23)	48.25 (0.27)	20.27 (0.09)	18.98 (0.01)	480.94 (2.93)
	Larch	43.68 (4.61)	46.87 (4.09)	21.60 (0.51)	24.72 (0.18)	564.12 (2.39)
Cycle 1	Pine	50.00 (0.66)	40.74 (0.08)	20.41 (0.33)	24.23 (1.47)	582.39 (26.91)
	Spruce	50.06 (0.04)	40.09 (0.11)	19.23 (0.09)	19.68 (1.12)	510.04 (28.80)
	Larch	50.06 (0.03)	40.66 (0.07)	20.33 (0.41)	23.56 (1.79)	568.79 (30.94)
Cycle 2	Pine	46.33 (3.96)	44.16 (3.71)	20.59 (0.17)	24.04 (0.96)	574.51 (14.20)
	Spruce	49.99 (0.38)	40.17 (0.12)	19.48 (0.04)	17.32 (0.63)	442.82 (13.79)
	Larch	44.13 (3.59)	46.64 (2.50)	22.13 (0.09)	24.30 (0.47)	537.35 (38.10)
Cycle 3	Pine	44.16 (4.34)	47.32 (4.34)	20.66 (0.03)	25.43 (0.11)	594.22 (1.74)
	Spruce	50.82 (0.94)	44.64 (0.33)	19.50 (0.35)	19.58 (0.28)	442.81 (13.75)
	Larch	47.47 (3.33)	43.46 (4.01)	20.02 (0.45)	23.81 (0.07)	580.72 (15.10)

^1^ SD—standard deviation.

**Table 2:** The standard deviation for every species of measured wood.

Modification	Mean (SD) ^1^, kg⋅m^−3^		
	Pine	Spruce	Larch
Native material	632.51 (4.42)	480.94 (3.48)	564.12 (2.36)
Cycle 1	582.39 (24.61)	510.04 (31.22)	568.79 (34.45)
Cycle 2	574.51 (14.16)	442.82 (15.27)	537.35 (38.34)
Cycle 3	594.22 (1.65)	442.81 (15.10)	580.72 (16.11)

^1^ SD—standard deviation.

**Table 3:** Tensile testing along the fibers of respective native materials.

Material	Mean Cross-Sectional Dimensions (SD) ^1^, mm		Mean Sample Destructive Force (SD) ^1^, kN	Mean Tensile Strength along the Fibers (SD) ^1^, MPa
	A	B	F	RrW
Pine	20.66 (0.30)	20.64 (0.38)	18.70 (0.68)	43.90 (2.47)
Spruce	20.40 (0.50)	20.24 (0.23)	21.62 (2.70)	52.36 (6.47)
Larch	20.88 (0.50)	20.92 (0.53)	23.89 (0.51)	54.76 (2.01)

^1^ SD—standard deviation.

**Table 4:** Tensile testing along the fibers of respective cycle 1 materials.

Material	Mean Cross-Sectional Dimensions (SD) ^1^, mm		Mean Sample Destructive Force (SD) ^1^, kN	Mean Tensile Strength along the Fibers (SD) ^1^, MPa
	A	B	F	RrW
Pine	21.05 (0.60)	21.09 (0.45)	24.16 (2.94)	54.48 (7.03)
Spruce	20.76 (0.37)	20.70 (0.53)	23.85 (2.95)	55.34 (5.05)
Larch	20.91 (0.12)	20.99 (0.22)	21.49 (1.54)	48.96 (3.78)

^1^ SD—standard deviation.

**Table 5:** Tensile testing along the fibers of respective cycle 2 materials.

Material	Mean Cross-Sectional Dimensions (SD) ^1^, mm		Mean Sample Destructive Force (SD) ^1^, kN	Mean Tensile Strength along the Fibers (SD) ^1^, MPa
	A	B	F	RrW
Pine	20.45 (0.16)	20.48 (0.20)	19.09 (1.65)	45.58 (4.03)
Spruce	20.25 (0.48)	20.57 (0.50)	22.67 (2.53)	54.42 (5.60)
Larch	20.88 (0.08)	20.95 (0.12)	21.41 (2.55)	48.96 (5.66)

^1^ SD—standard deviation.

**Table 6:** Tensile testing along the fibers of respective cycle 3 materials.

Material	Mean Cross-Sectional Dimensions (SD) ^1^, mm		Mean Sample Destructive Force (SD) ^1^, kN	Mean Tensile Strength along the Fibers (SD) ^1^, MPa
	A	B	F	RrW
Pine	20.45 (0.12)	20.27 (0.28)	20.69 (2.11)	49.88 (4.88)
Spruce	20.11 (0.24)	20.11 (0.28)	23.02 (2.94)	56.88 (6.80)
Larch	20.64 (0.33)	20.37 (0.14)	20.63 (2.22)	49.04 (4.94)

^1^ SD—standard deviation.

**Table 7:** Effect of the number of pine soaking cycles used on compressive strength values.

Material	Tensile Strength along the Fibers, RrW [MPa]		
	Pine	Spruce	Larch
Native	43.90171 ^a^	52.38892 ^c^	48.95715 ^d^
Cycle 1	54.48021 ^b^	56.87965 ^c^	54.76477 ^d^
Cycle 2	45.57737 ^a^	54.42522 ^c^	48.97611 ^d^
Cycle 3	49.89244 ^a.b^	55.35582 ^c^	49.03944 ^d^

^a, b, c, d^—homogenous group.

**Table 8:** The percentage share of ash content.

Material	Ash Content Native, %	Ash Content Cycle 1, %	Ash Content Cycle 2, %	Ash Content Cycle 3, %
Pine	0.51	0.74	0.97	0.68
	0.34	0.68	0.79	0.74
	0.40	0.91	0.91	0.85
Spruce	0.62	0.68	0.68	0.45
	0.51	0.74	0.69	1.02
	0.45	0.65	0.61	0.90
Larch	0.51	0.46	0.68	0.74
	0.40	0.68	0.85	0.80
	0.40	0.85	0.68	0.74

## Data Availability

Not applicable.
